# Frequency and predictors for early-achieved lupus low disease activity state in systemic lupus erythematosus patients treated with telitacicept or belimumab: A real-life, single-center observational study

**DOI:** 10.3389/fimmu.2024.1423035

**Published:** 2024-06-14

**Authors:** Cuiling Fan, Tao Yang, Songyuan Zheng, Xiaozhong Liao, Ruixia Xie, Shixian Chen, Juan Li

**Affiliations:** ^1^ Department of Rheumatology and Immunology, Nanfang Hospital, Southern Medical University, Guangzhou, China; ^2^ Department of Traditional Chinese Internal Medicine, School of Traditional Chinese Medicine, Southern Medical University, Guangzhou, China

**Keywords:** systemic lupus erythematosus, lupus low disease activity state, belimumab, telitacicept, treat-to-target, predictor

## Abstract

**Objective:**

To collect real-world data regarding the attainment of the early-achieved lupus low disease activity state (LLDAS) in systemic lupus erythematosus (SLE) patients receiving telitacicept or belimumab treatment, and identify factors predictive of target achievement.

**Methods:**

Eighty-seven SLE patients who received telitacicept (N=42) or belimumab (N=45) were retrospectively reviewed in this observational study. Clinical and laboratory data, disease activity assessment, and glucocorticoid dosage were collected for analysis. Achieving LLDAS at least once within 24 weeks post-treatment was considered as early-achieved LLDAS. Multivariate regression was used to assess baseline predictive variables for early-achieved LLDAS. Subgroup analysis and interaction tests were also performed to examine the robustness of the results across different sets of baseline characteristics. Prognostic stratification for early-achieved LLDAS was established based on the identified risk factors.

**Results:**

During the 24-week follow-up period, LLDAS was achieved by at least one time in 49.43% (43/87) of the patients, with sustained achievement through week 24 observed in 36 out of these 43 patients (83.27%). Multivariate analysis revealed that early achievement of LLDAS was particularly observed in patients with higher baseline lymphocyte counts [HR=1.79, 95% CI (1.19–2.67), P=0.005]and serum albumin levels [HR=1.06, 95% CI (1.003–1.12), P=0.039]. Conversely, hematological involvement [HR=0.48, 95% CI (0.24–0.93), P=0.031] predicted lower attainment of early-achieved LLDAS. The use of telitacicept was associated with a reduced risk of failing to attain early achievement of LLDAS [HR=2.55, 95% CI (1.36–4.79), P=0.004]. Subgroup analyses and interaction tests showed a stable relationship between the telitacicept use and LLDAS achievement. The results remained consistent across all subgroup analyses. Significant differences (P<0.001) were observed in the Kaplan-Meier estimates for LLDAS among risk groups based on the number of identified risk factors.

**Conclusion:**

The achievement of LLDAS is attainable in the management of SLE patients undergoing treatment with telitacicept or belimumab in real-life clinical practice. Baseline lymphocyte counts, serum albumin levels, hematological involvement and the use of telitacicept serve as robust predictors for early-achieved LLDAS, helping to identify patients who are likely to benefit on the treatment.

## 1 Introduction

Systemic lupus erythematosus (SLE) is a heterogeneous autoimmune disease characterized by a myriad of manifestations, unpredictable periods of heightened disease activity and variability in response to conventional immunosuppressants, which make it challenging for treatment decisions and prognostication (1). For SLE patients, the treat-to-target approach is a therapeutic strategy that aims for personalized, precise, and systematic treatment to better control disease activity, improve clinical symptoms, and achieve clinical remission. Within the framework of defining disease modification objectives, lupus low disease activity state (LLDAS), along with glucocorticoid reduction and flare prevention, can be considered as crucial targets (2). Attaining LLDAS while minimizing treatment-related toxicities has been associated with reduced disease flares and organ damage accumulation in SLE patients (3–5). Measuring LLDAS in SLE can demonstrate significant effects of an intervention on modifying the course of the disease. Previous studies have shown that failure to achieve LLDAS within 6 months of initial therapy is linked to early damage accrual and long-term prognosis. Therefore, investigating prognostic stratification for predicting early achievement of LLDAS could aid in facilitating initial evaluation and treatment decisions for SLE patients (6, 7). The approved treatments for SLE, telitacicept and belimumab, exert their therapeutic effects through the inhibition of B-cell activating factor (BAFF) and a proliferation-inducing ligand (APRIL). They were anticipated to achieve the treatment target more expeditiously compared to standard therapy. The efficacy of each treatment has been demonstrated in clinical trials, showing its effectiveness in managing disease activity, reducing glucocorticoid exposure, and minimizing damage accumulation in patients with SLE (8–11). The post-analysis of randomized clinical trials on belimumab, including BLISS-52 and BLISS-76, has substantiated its efficacy in achieving LLDAS, thereby establishing LLDAS as a viable endpoint for SLE clinical trials, particularly when retrospectively applied (12). A real-world observational study demonstrated that lower prednisone doses at baseline were significantly associated with achieving LLDAS at 12 months, even after adjusting for potential confounding factors (13). The efficacy of telitacicept in achieving an SRI-4 response has been reported as promising (14). However, there is limited available information regarding the frequency and predictors of early attainment of LLDAS in routine clinical practice among patients with SLE. Furthermore, due to the absence of a head-to-head randomized controlled trial comparing belimumab and telitacicept, the differential roles of these agents in achieving LLDAS remain unclear. Herein, we present an investigation into the frequency and predictors for early-achieved LLDAS in patients receiving BAFF/APRIL inhibitors. Additionally, a comparison was made between telitacicept and belimumab regarding the early achievement of LLDAS. Finally, a prognostic stratification for early-achieved LLDAS was established based on identified risk factors. This study provides valuable evidence to inform treatment practices.

## 2 Materials and methods

### 2.1 Study patients

The study was conducted at the Rheumatology Department of Nanfang Hospital, Southern Medical University, and obtained approval from the Central Ethics Committee of Nanfang Hospital, Southern Medical University (NFEC2023490). Informed consent was obtained from all participants. Retrospective inclusion criteria consisted of patients who met the 1997 American College of Rheumatology (ACR) criteria for SLE and received telitacicept or belimumab for a minimum duration of 24 weeks between 2021 and 2023 at our Rheumatology Department. Patients who had been exposed to other biologics (such as telitacicept, belimumab or rituximab) within six months prior to cohort entry, those with active infections, current pregnancy, or a history of malignancy were excluded from the analysis.

### 2.2 Data collection

LLDAS attainment for the first time within 24 weeks was analyzed as the primary endpoint for all the patients. Early-achieved LLDAS was defined as achieving LLDAS within 24 weeks after treatment initiation. Clinical features, laboratory data, serological results (complements and Anti-dsDNA positivity), disease activity and treatments were collected at baseline and during each subsequent follow-up visit. Disease activity was evaluated by SLE Disease Activity Index 2000 (SLEDAI-2K), Clinical SLEDAI-2K (SLEDAI-2K excluding Anti-dsDNA and complement scores) and the Physician’s Global Assessment of disease activity (PGA). The achievement of LLDAS must satisfy the following five sub-criteria, as previously stated (2): ① SLEDAI-2K ≤4, with no evidence of activity in any major organ; ② absence of new disease activity features compared to the previous assessment; ③ PGA ≤ 1; ④ current daily prednisone dosage ≤7.5 mg; ⑤ standard maintenance dosages of immunosuppressive drugs and approved biologics allowed.

### 2.3 Subgroups

Patients were divided by baseline BAFF/APRIL inhibitors use into telitacicept and belimumab subgroups. The early achievement of LLDAS was compared between telitacicept and belimumab subgroups. For assessment of the consistency of the association between telitacicept use and early achievement of LLDAS, relative subgroup analyses and interaction tests were conducted based on baseline characteristics selected by clinical significance.

### 2.4 Statistical analysis

Continuous data were presented as median (interquartile range, IQR) and tested using the Kruskal-Wallis tests. Categorical variables were described by frequencies and percentages and tested with either Pearson’s chi-square test or Fisher’s exact test. Survival analyses utilized Kaplan-Meier methods, with differences in achieving LLDAS as the primary endpoint assessed via log-rank test. Univariate and multivariate analyses employed Cox proportional hazards model to derive hazard ratios (HRs) along with corresponding 95% confidence intervals (CIs). The variables with P-values below 0.05 or potential confounders identified in the univariate analysis were included in the subsequent multivariate analysis to determine independent risk factors. To address the potential issue of multicollinearity and over-fitting, we employed the “glmnet” package in R software to perform least absolute shrinkage and selection operator (LASSO) Cox regression analysis. This approach enabled us to identify the most significant factors associated with the outcome of interest. In LASSO Cox model, we applied an L1 penalty to accurately shrink certain regression coefficients towards zero. Additionally, a 10-fold cross-validation was performed to determine the optimal value of log(λ), which serves as a tuning parameter controlling the degree of shrinkage. To identify independent prognostic factors for LLDAS, all factors with non-zero coefficients from the LASSO analysis were incorporated into a multivariable Cox regression analysis. Subgroup analyses were conducted using stratified Cox regression models, while interaction tests were employed to assess consistency across various subgroups. Statistical analyses were performed using R software (version 4.2.3), SPSS software (version 23.0). A P-value < 0.05 was considered to indicate statistical significance.

## 3 Results

### 3.1 Patient characteristics

A total of 87 patients with SLE who received telitacicept (N=42) and belimumab (N=45) were included and analyzed. As presented in **Table 1**, median (IQR) age of the study participants was 30 (22–36) years, with a majority of them being female (87.36%). The median disease duration was 44 months, ranging from 9 to 129.5 months. At the beginning of the study, all patients were in active stage of SLE. The median SLEDAI-2K, Clinical SLEDAI-2K, and PGA were recorded as follows: 8 (5–14), 6 (4–10), and 0.90 (0.55–1.60), respectively. More than 70% of the patients had received treatments within three months prior to initiating BAFF/APRIL inhibitors. Most of the enrolled patients were administered prednisone and immunosuppressive agents during the follow-up period. In the overall population, mucocutaneous involvement (63.22%), hematological involvement (48.28%), and renal involvement (41.38%) were identified as the most prevalent clinical manifestations. Anti-dsDNA was detected in 57 (65.52%) patients, while hypocomplementemia was observed in 46 (52.87%) patients.

**Table 1:** Demographic, clinical and serological characteristics at baseline.

Variable	Overall^1^	LLDAS^1^	N-LLDAS^1^	P value
	N=87	N=43	N=44	
Gender(female)	76 (87.36)	36 (83.70)	40 (90.90)	0.313
Age(years)	30.00 (22.00-36.00)	30.00 (22.00-36.00)	31 (22.00-36.00)	0.885
Age at diagnosis(years)	22.00 (16.00-28.50)	21.00(16.00-30.00)	22.00 (16.00-28.00)	0.782
Disease duration(years)	44.00 (9.00-129.50)	30.00 (6.00-125.00)	69.50 (12.25-134.50)	0.283
Disease activity				
SLEDAI-2K	8.00 (5.00-14.00)	6.00 (4.00-12.00)	10.00 (6.25-14.75)	**0.017**
Clinical SLEDAI-2K	6.00 (4.00-10.00)	6.00 (3.00-9.00)	8.00 (4.00-12.00)	**0.032**
PGA	0.90 (0.55-1.60)	0.80(0.40-1.60)	1.20 (0.63-2.00)	**0.038**
Organ involvement				
Gastrointestinal	4 (4.60)	2 (4.65)	2 (4.55)	0.981
Neurological	7 (8.05)	5 (11.63)	2 (4.55)	0.225
Cardiovascular/respiratory	10 (11.49)	2 (4.65)	8 (18.18)	0.089
APS	12 (13.79)	4 (9.30)	8 (18.18)	0.23
Musculoskeletal	32 (36.78)	17 (39.53)	15 (34.09)	0.599
Mucocutaneous	55 (63.22)	28 (65.12)	27 (61.36)	0.717
Hematological	42 (48.28)	15 (34.88)	27 (61.36)	**0.013**
Renal	36 (41.38)	12 (27.91)	24 (54.55)	**0.012**
Constitutional	23 (26.44)	12 (27.91)	11 (25.00)	0.759
Serosal	9 (10.34)	4 (9.30)	5 (11.36)	0.752
Serologic				
Hypocomplementemia	46 (52.87)	18 (41.86)	28 (63.64)	**0.042**
C_3_(g /L)	0.71 (0.54-0.95)	0.81 (0.58-1.06)	0.68 (0.52-0.88)	0.052
C_4_(g /L)	0.14 (0.08-0.21)	0.15 (0.10-0.23)	0.11 (0.07-0.18)	0.136
Anti-dsDNA	57 (65.52)	25 (58.14)	32 (72.73)	0.152
Anti-dsDNA(μ /mL)	128.33 (25.67-420.11)	90.94 (19.12-375.03)	254.74 (47.95-548.69)	0.118
Medications				
Untreated	26 (29.89)	12 (27.91)	14 (31.82)	0.69
BAFF/APRIL inhibitors				**0.007**
Belimumab	45 (51.72)	16 (37.21)	29 (65.91)	
Telitacicept	42 (48.28)	27 (62.79)	15 (34.09)	
Daily prednisone (mg/d)	20.00 (10.00-45.00)	15.00 (5.00-45.00)	22.50 (10.00-45.00)	0.237
Mycophenolate	49 (56.32)	24 (55.81)	25 (57)	0.925
Azathioprine	6 (6.90)	3 (6.98)	3 (6.8)	0.977
Methotrexate	3 (3.45)	2 (4.65)	1 (2.3)	0.543
Leflunomide	4 (4.60)	2 (4.65)	2 (4.5)	0.981
Tacrolimus	5 (5.75)	1 (2.33)	4 (9.1)	0.175
Ciclosporin	16 (18.39)	7 (16.28)	9 (20.00)	0.615
HCQ	83 (95.40)	39 (90.70)	44 (100.00)	0.055
ANA	86 (98.85)	42 (97.67)	44 (100.00)	0.309
ANA(μ /mL)	265.99 (73.24-407.65)	300.00 (97.66-407.65)	203.04 (70.21-411.1)	0.634
Anti-SSA	46 (52.87)	25 (58.14)	21 (47.73)	0.331
Anti-SSB	13 (14.94)	10 (23.26)	3 (6.82)	**0.032**
Anti-Sm	31 (35.63)	15 (34.88)	16 (36.36)	0.885
Anti-U1RNP	55 (63.22)	28 (65.12)	27 (61.36)	0.717
ARPA/Rib-P	30 (34.48)	13 (30.23)	17 (38.64)	0.41
AnuA	42 (48.28)	17 (39.53)	25 (56.82)	0.107
AHA	41 (47.13)	19 (44.19)	22 (50.00)	0.587
AMAM2	12 (13.79)	7 (16.28)	5 (11.36)	0.506
Anti-Ro52	41 (47.13)	25 (58.14)	16 (36.36)	**0.042**
Anti-β2GP1	19 (21.84)	8 (18.60)	11 (25.00)	0.47
ACA	10 (11.49)	4 (9.30)	6 (13.64)	0.526
Proteinuria	39 (44.83)	15 (34.88)	24 (54.55)	0.065
WBC(× 10^9^ /L)	5.39 (4.11-7.04)	5.56 (4.36-7.51)	4.52 (3.47-6.44)	**0.015**
Lymphocyte counts(× 10^9^ /L)	1.37 (0.91-1.90)	1.55 (1.24-2.28)	1.04 (0.68-1.56)	**<0.001**
Neutrophils counts(× 10^9^ /L)	3.22 (2.26-4.48)	3.62 (2.43-4.96)	3.12 (2.18-4.09)	0.14
Monocyte counts(× 10^9^ /L)	0.42 (0.27-0.57)	0.44 (0.29-0.63)	0.42 (0.24-0.54)	0.13
HGB(g /L)	118.00 (98.00-131.50)	120.00 (104.00-133.00)	107.50 (97.00-130.75)	0.132
PLT(× 10^9^ /L)	22 (176.50-267.50)	232.00 (167.00-294.00)	219.00 (180.25-258.75)	0.599
IgA(g /L)	2.52 (1.92-3.59)	2.52 (1.91-3.65)	2.52 (1.9-3.58)	0.872
IgM(g /L)	0.84 (0.61-1.06)	0.85 (0.63-1.32)	0.83 (0.61-1.04)	0.569
IgG(g /L)	13.26 (10.07-16.37)	13.49 (10.86-16.99)	12.85 (8.81-16.29)	0.172
CH_50_(g /L)	33.80 (21.00-44.00)	36.00 (21.00-49.00)	31.50 (17.25-38.50)	0.099
ESR(mm /H)	27.00 (12.00-43.50)	20.00 (8.00-39.00)	31.50 (14.25-58.50)	**0.047**
CRP(mg /L)	1.66 (0.61-3.79)	1.13 (0.5-5.13)	1.88 (0.84-3.75)	0.558
Serum albumin(g /L)	35.80 (31.65-39.85)	37.90 (33.30-41.00)	34.05 (31.03-38.78)	**0.026**
Globulin(g /L)	27.80 (24.25-32.90)	28.50 (25.30-32.70)	27.65 (23.05-33.93)	0.513

LLDAS, lupus low disease activity state; N-LLDAS, non-LLDAS; Clinical SLEDAI-2K, SLEDAI-2K excluding Anti-dsDNA and complement scores; PGA, Physician’s Global Assessment; APS, anticardiolipin syndrome; C_3_, complement C_3_; C_4_, complement C_4_; HCQ, hydroxychloroquine; WBC, white blood cell; HGB, hemoglobin; PLT, platelet; Ig, immunoglobulin; CH_50_, 50% hemolytic unit of complement; CRP, C-reactive protein; ESR, erythrocyte sedimentation rat; ^1^N (%) or median (IQR); Values in bold indicate that P values < 0.05.

### 3.2 Attainment of LLDAS

**Figure 1:**
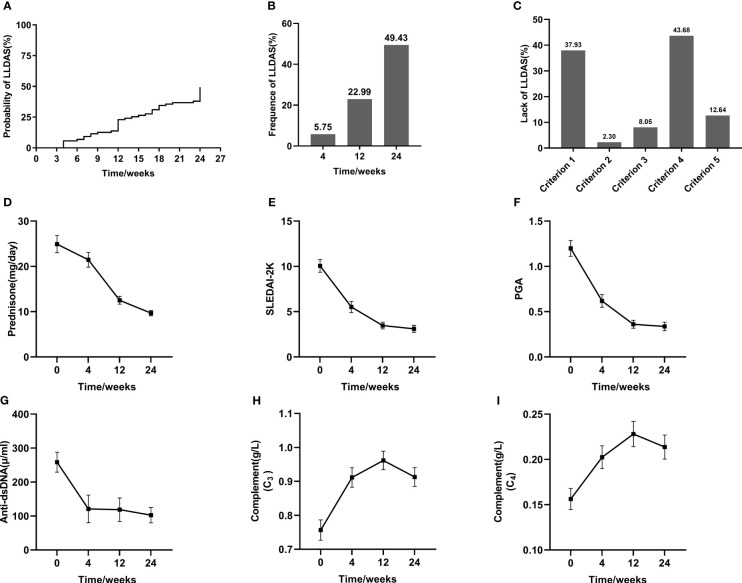
Achievement of LLDAS and the improvement of SLE parameters after BAFF/APRIL inhibitors treatment. Kaplan-Meier estimations of the accumulated **(A)** and cross-sectional **(B)** frequency of LLDAS during a 24-week follow-up period. Analysis of lack of LLDAS achievement **(C)**. Changes of individual parameters throughout the follow-up period **(D–I)**.

The administration of BAFF/APRIL inhibitors resulted in significant reductions in SLEDAI-2K and PGA scores over the course of treatment, starting as early as 4 weeks. Moreover, as complement levels increased, there was a concomitant decrease in Anti-dsDNA concentration, along with a notable reduction in daily prednisone dosage from baseline to the endpoint (**Figures 1D-I**). The overall cumulative probabilities of achieving LLDAS were presented in **Figures 1A, B**. Attainment of LLDAS was 5.75% and 22.99% at weeks 4 and 12, respectively. By week 24, LLDAS was achieved at least once by 49.43% (N=43) of patients. 7 Out of 43 (16.28%) patients failed to get persistent LLDAS achieved during follow-up period. The major causes for the loss of LLDAS in these 7 patients included the emergence of disease activity manifestations (3 with cutaneous, 2 with articular, 1 with hematological involvement, and 1 with both cutaneous and fever manifestations), drug treatment intolerance in 2 patients, and a newly discovered positivity for Anti-dsDNA or low complement in one patient. Focusing on unmet criteria for LLDAS: 33 (37.93%) patients was active in major organ or did not have SLEDAI-2K ≤4; 2 (2.30%) patients experienced new manifestation; 7 of them (8.05%) had PGA > 1; 38 (43.68%) patients were not on prednisolone ≤7.5 mg/day; and 11 (12.64%) patients did not meet the criterion 5 (standard maintenance dosages of immunosuppressive drugs and approved biologics allowed) (**Figure 1C**).

### 3.3 Factors associated with early-achieved LLDAS

We divided the SLE patients into two groups based on their achievement of LLDAS, namely the LLDAS group and the N-LLDAS group. The LLDAS group exhibited significantly lower disease activity (P<0.05) and erythrocyte sedimentation rate (ESR) (P=0.047), as well as higher levels of serum albumin, white blood cell (WBC) count, and lymphocyte counts (P=0.026, P=0.015, and P<0.001 respectively). Additionally, they had a lower incidence of hematological involvement (P=0.013), renal involvement (P=0.012), hypocomplementemia (P=0.042), and a higher utilization of telitacicept treatment option (P=0.007). Moreover, there was an increased proportion of Anti-Ro52 positive cases (P=0.042) and Anti-SSB positive cases (P=0.032) (**Table 1**).

**Figure 2:**
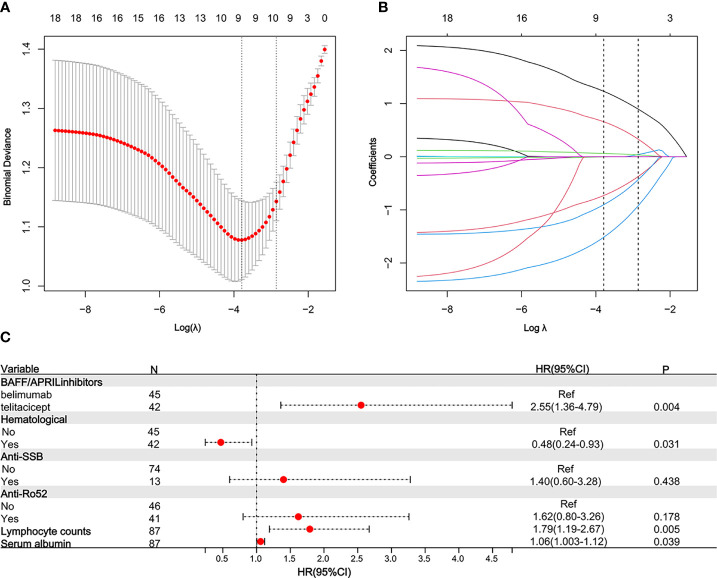
Predictors for LLDAS selected by LASSO Cox regression and multivariable Cox regression model. 10-fold cross-validation to select the optimal tuning parameter log (λ). Dotted vertical lines were drawn at the optimal values by using the minimum criteria and the 1 SE of the minimum criteria (the 1-SE criteria) **(A)**. LASSO coefficient profiles of 12 features. The optimal lambda produces 6 features of nonzero coefficients **(B)**. The LASSO Cox regression model identified 6 predictors with non-zero coefficients. A forest plot visually depicting the aforementioned multivariate Cox regression outcomes **(C)**. HR, Hazard ratios; CI, confidence interval; LLDAS, lupus low disease activity state.

**Figure 3:**
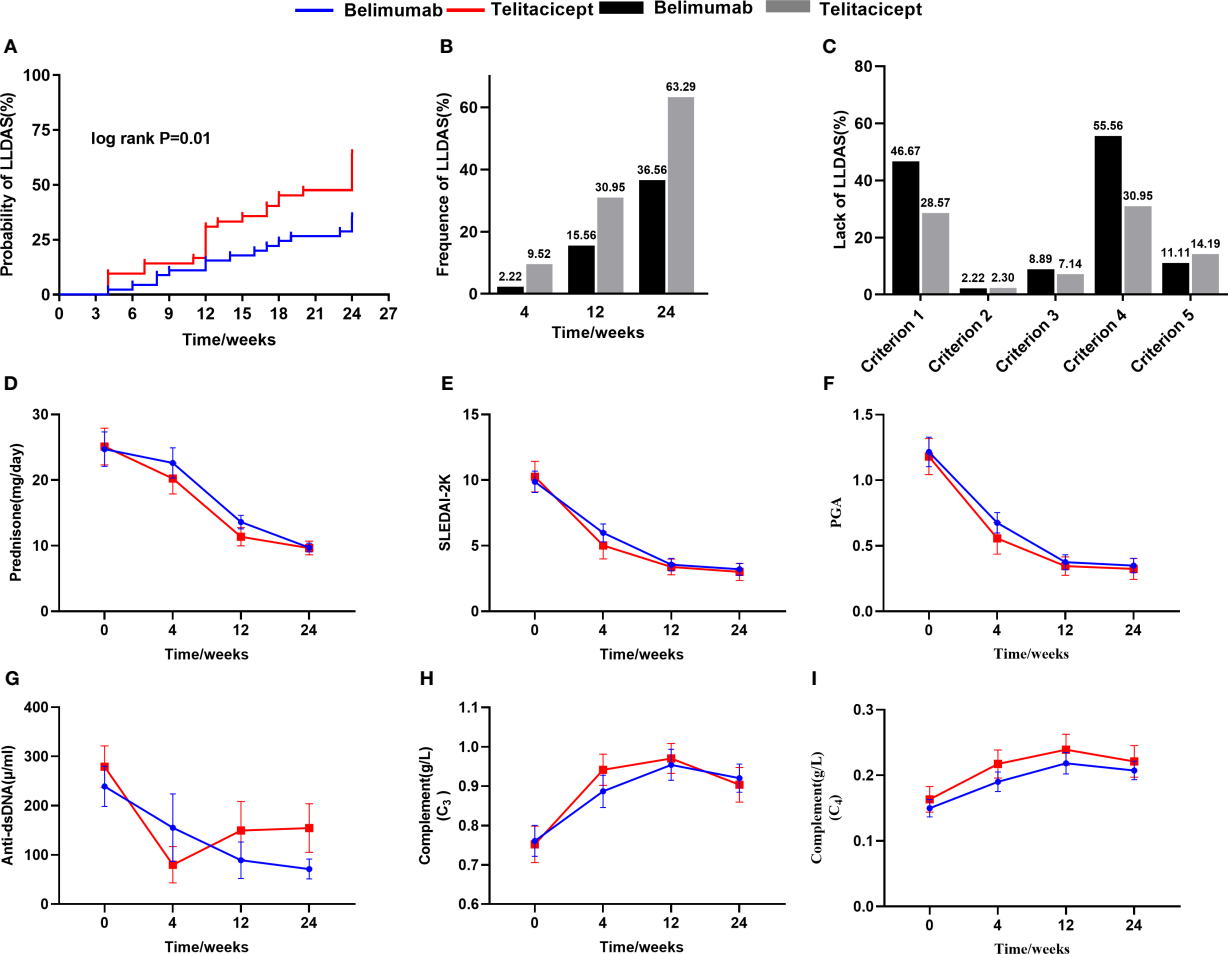
The comparison of the achievement of LLDAS and the improvement of SLE parameters between belimumab and telitacicept treatment. Kaplan-Meier curves showing LLDAS achievement in patients after belimumab or telitacicept treatment after a 24 weeks follow-up **(A)**. The comparison of cross-sectional frequency of LLDAS between belimumab and telitacicept treatment **(B)**. The lack of LLDAS achievement of belimumab and telitacicept **(C)**. The difference of improvement of SLE parameters after treatment between belimumab and telitacicept **(D-I)**.

The univariable analysis of early-achieved LLDAS using Cox regression was presented in **Table 2**. The following factors were found to have statistical significance (P<0.05): Baseline SLEDAI-2K, Clinical SLEDAI-2K, types of BAFF/APRIL inhibitor, serum albumin and complement C_3_ levels, WBC and lymphocyte counts, hematological involvement, renal involvement, hypocomplementemia, Anti-Ro52 positivity and Anti-SSB positivity. To address the issues of multicollinearity and over-fitting in the regression analysis, we employed a LASSO Cox regression model by including variables with a significance level of P<0.05 (**Figures 2A, B**). Subsequently, at the optimal lambda value that minimizes bias, we identified six indicators associated with early achievement of LLDAS that had non-zero coefficients (**Supplementary Table S1**). These indicators encompassed types of BAFF/APRIL inhibitor, serum albumin level and lymphocyte counts, hematological involvement, Anti-Ro52 positivity, and Anti-SSB positivity; all of which were incorporated into the multivariate Cox regression model. Ultimately, the results demonstrated a significant correlation between baseline use of telitacicept [HR=2.55, 95% CI (1.36–4.79), P=0.004] and early achievement of LLDAS. Other independent predictors include lymphocyte counts [HR=1.79, 95% CI (1.19–2.67), P=0.005], serum albumin level [HR=1.06, 95% CI (1.003–1.12), P=0.039], and hematological involvement [HR=0.48, 95% CI (0.24–0.93), P = 0.031] (**Figure 2C**). Utilizing the optimal cut-off values for lymphocyte counts (0.95×10^9^/L) and serum albumin levels (33.25g/L), a total of 23 patients (73.56%) were categorized into the low lymphocyte count group while 31 patients (35.63%) fell into the low serum albumin group within this study cohort. Kaplan-Meier estimations based on predictive factors demonstrated a statistically significant increase in the likelihood of achieving LLDAS in patients with low lymphocyte count, low serum albumin level or hematological involvement (**Supplementary Figures S1A-C**). And patients receiving telitacicept add on had a significantly high probability of achieving LLDAS compared to those receiving belimumab (**Figure 3A**).

**Table 2:** Univariable and multivariable cox regression estimating the risk factors of early-achieved LLDAS.

Variable	Univariate		Multivariate^1^	
	HR (95% CI)	P value	HR (95% CI)	P value
Clinical SLEDAI-2K	0.92 (0.84-0.999)	0.046		
SLEDAI-2K	0.92 (0.86-0.995)	0.035		
**Hypocomplementemia**				
No	Ref			
Yes	0.41 (0.17-0.98)	0.044		
**Renal**				
No	Ref			
Yes	0.32 (0.13-0.79)	0.013		
WBC	1.22 (1.01 -1.47)	0.035		
C_3_	5.77 (1.14-29.17)	0.034		
**Anti-SSB**				
No	Ref		Ref	
Yes	4.14 (1.05-16.19)	0.042	1.40 (0.60-3.28)	0.438
**BAFF/APRIL inhibitors**				
belimumab	Ref		Ref	
telitacicept	3.26 (1.36-7.85)	0.008	2.55 (1.36-4.79)	0.004
**Hematological**				
No	Ref		Ref	
Yes	0.34 (0.14-0.81)	0.015	0.48 (0.24-0.93)	0.031
**Anti-Ro52**				
No	Ref		Ref	
Yes	2.43 (1.03-5.76)	0.044	1.62 (0.80-3.26)	0.178
**Lymphocyte counts**	4.11 (1.90-8.90)	<0.001	1.79 (1.19-2.67)	0.005
**Serum albumin**	1.09 (1.01-1.17)	0.023	1.06 (1.003-1.12)	0.039

HR=Hazard ratios; CI=confidence interval; C_3_=complement C_3_; WBC=white blood cell; ^1^Multivariate Cox regression model included the variables selected by LASSO Cox regression, which was write in bold.

### 3.4 Subgroup analyses

**Figure 4:**
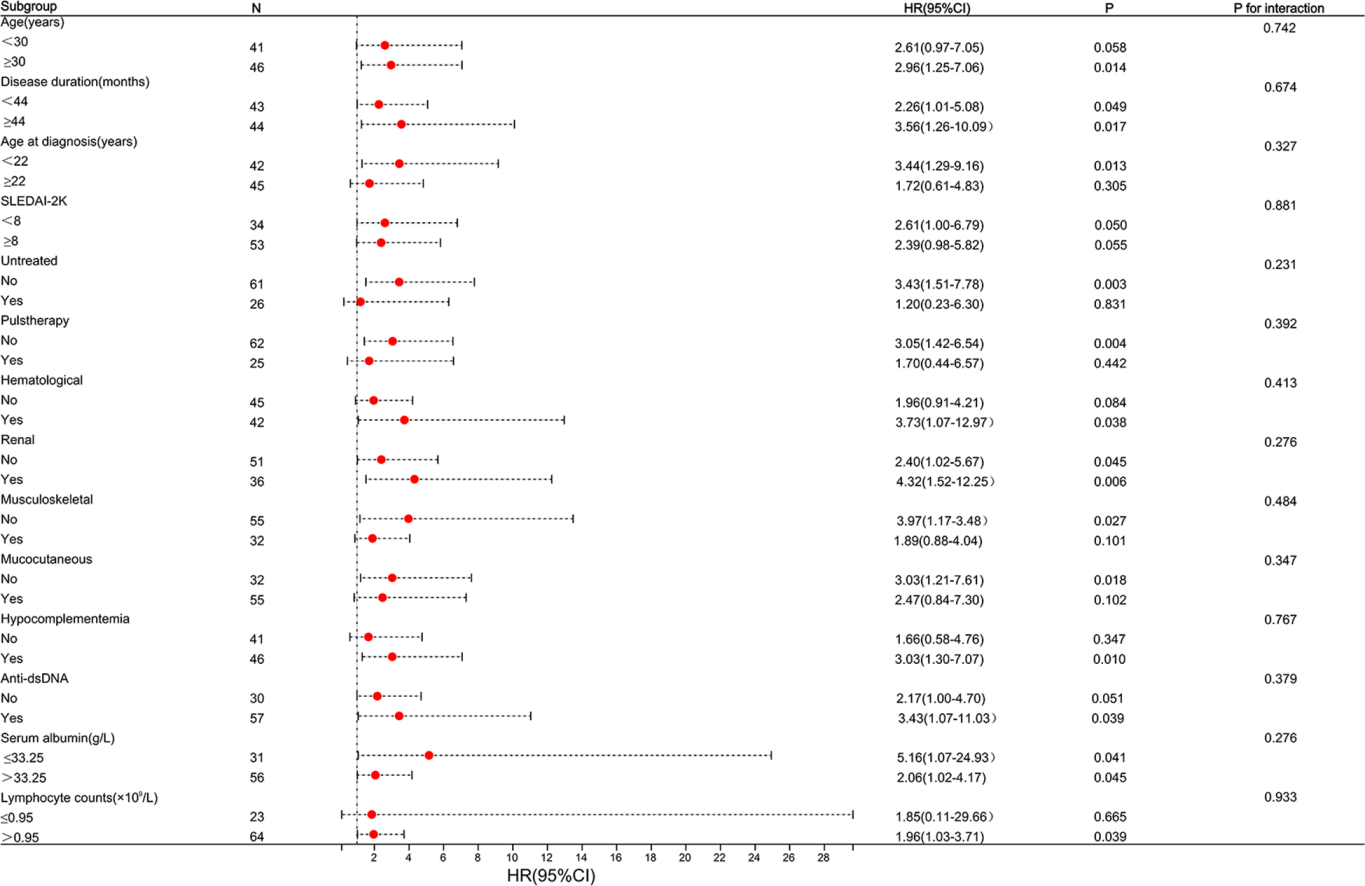
Subgroup analysis of association between types of BAFF/APRIL inhibitors and LLDAS achievement. Horizontal lines represent 95% confidence intervals. HR, Hazard ratios; CI, confidence interval. The cut-off for continuous variables was determined according to our population median value or the optimal cut-off values. Above model adjusted for hematological involvement, lymphocyte counts and serum albumin level. In each case, the model is not adjusted for the stratification variable.

According to the results, it appears that patients with SLE derive greater benefits from telitacicept compared to belimumab in early-achieved LLDAS. Subgroup analyses and interaction tests were conducted to assess the consistency of the association between telitacicept use and early achievement of LLDAS in 14 subgroups defined based on baseline characteristics. The subgroup analyses were performed using stratified Cox regression models, with adjustments made for other independent predictors such as hematological involvement, lymphocyte counts, and serum albumin levels. Importantly, subgroup analyses demonstrated the consistent positive effect of telitacicept use on early achievement of LLDAS. This correlation remained stable across all subgroups without significant impact (P>0.05) (**Figure 4**). For the two subgroups divided by BAFF/APRIL inhibitors use (telitacicept versus belimumab), there were no significant differences observed in patient demographic and clinical characteristics at baseline between the two groups, except for the proportion of serosal involvement (P=0.013), ACA positivity (P=0.033) and CRP level (P=0.016) (**Supplementary Table S2**). The frequency of early-achieved LLDAS attainment over time among patients undergoing telitacicept and belimumab therapy gradually increase. Patients who received telitacicept demonstrated a higher probability of attaining early-achieved LLDAS at 64.29% (27/42) compared to those who received belimumab with a probability of 35.56% (16/45) (**Figure 3B**). Further analysis of the frequence of LLDAS sub-criteria between these two groups revealed that patients receiving telitacicept exhibited a higher likelihood of meeting criterion 1 and criterion 4 (**Figure 3C**). Additionally, there were no significant differences observed between the two groups in terms of changes in SLEDAI-2K, PGA, complement (C_3_ and C_4_), Anti-ds DNA levels, and daily prednisone dosage during the follow-up period (**Figures 3D–I**). When comparing the baseline characteristics of the LLDAS and N-LLDAS groups in the telitacicept group, we observed a lower baseline WBC count and lymphocyte count in the N-LLDAS group. Additionally, there was a higher IgG level and ANA quantification in the LLDAS group (**Supplementary Table S3**). In contrast, within the belimumab group, we found that N-LLDAS patients at baseline exhibited high disease activity (SLEDAI-2K, clinical-SLEDAI 2K), higher renal involvement and hematological involvement rates, as well as a greater proportion of hypocomplementemia and AnuA positivity. Furthermore, these patients had a lower baseline lymphocyte count (**Supplementary Table S4**). The individual analysis reveals that in the belimumab group, there were 17 patients who did not meet both criterion 1 and criterion 4 for LLDAS (**Supplementary Figure S2**). Similarly, these patients exhibited prominent clinical features, including high disease activity, decreased levels of serum albumin, complement and lymphocyte counts, renal involvement in 64.71% (11/17) of cases, and hematological involvement in 47.06% (8/17) of cases.

### 3.5 Prognostic stratification for early-achieved LLDAS

**Figure 5:**
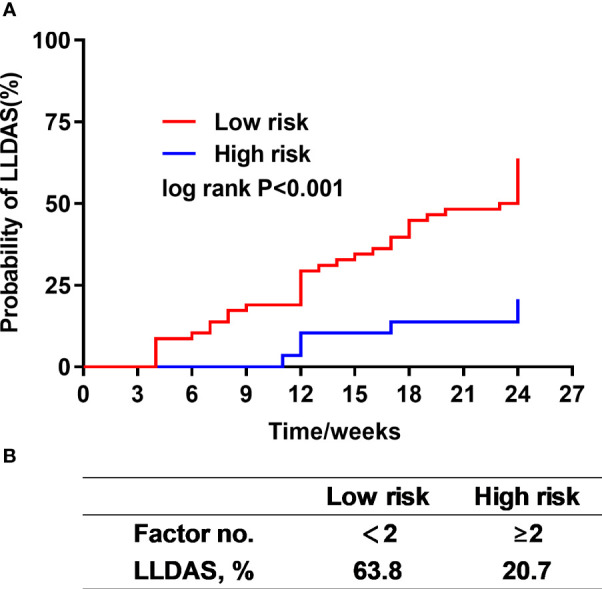
The prognostic stratification for early-achieved LLDAS according to independent predictors in the cohort. The Kaplan-Meier estimations of LLDAS incidence were demonstrated among the two risk groups **(A)**. The probability of achieving LLDAS declined with an increasing number of risk factors **(B)**.

The predictor-based risk classifier was established based on three potential risk factors associated with the failure to attain early-achieved LLDAS, including low lymphocyte counts, low serum albumin levels, and hematological involvement. The risk of failing to attain early-achieved LLDAS in each patient was determined based on the presence of these three risk factors. Subsequently, the patients were classified into a low-risk group (<2 factors) or a high-risk group (≥2 risk factors). In the entire cohort, the probability of reaching LLDAS for patients in the low-risk and high-risk groups was 63.8% and 20.7%, respectively (log-rank P<0.001) (**Figure 5**). After conducting stratified analysis based on the use of BAFF/APRIL inhibitors, significant differences in estimations of LLDAS among the risk groups remained evident. In patients treated with belimumab, the probability of attaining early-achieved LLDAS was 50% and 6.7% for patients in the low-risk group and high-risk group, respectively (log-rank P=0.007) (**Supplementary Figures S3A, B**). For patients treated with telitacicept, the probability of early-achieved LLDAS was 78.6% and 35.7% for those in the low-risk group and high-risk group, respectively (log-rank P=0.009) (**Supplementary Figures S3C, D**).

### 3.6 Adverse Events

17 (18.39%) patients had 19 adverse events (AEs) reported (**Supplementary Table S2**). One patient experienced cholecystitis. The remaining adverse events consisted of 5 cases of lower urinary tract infections, 6 instances of herpes zoster reactivations, 3 cases of upper respiratory infections, and 2 cases of herpes simplex virus infections. Additionally, 2 patients developed pneumonia, successfully achieved full recovery after receiving antimicrobial therapy.

## 4 Discussion

LLDAS is key outcome measurement for the treat-to-target strategy in SLE. Early-achieved LLDAS might be associated with long-term disease status of SLE (7, 15). However, there is currently limited data available regarding the time required to attain LLDAS and the clinical factors that influence its achievement, particularly in SLE patients on biologics treatment. Herein, we present the findings of a single-center, real-world study conducted on active SLE patients who received treatment with BAFF/APRIL inhibitors, specifically belimumab or telitacicept. And the attainment of the early-achieved LLDAS, defined as achieving LLDAS at least once within 24 weeks post-treatment, in clinical practice was demonstrated. Our results indicate that the administration of belimumab and telitacicept in patients with active SLE helps to attain early-achieved LLDAS by effectively reducing disease activity and minimizing glucocorticoid usage. Baseline low lymphocyte counts, low serum albumin level and hematological involvement were identified as risk factors for failure to achieve LLDAS. Furthermore, the use of telitacicept was significantly associated with early attainment of LLDAS. This positive effect was consistent across all subgroups considered and after careful adjustments. Simultaneously, the stratification of prognostic based on the number of risk factors effectively predicts early-achieved LLDAS. This emphasizes the importance of considering both the frequency and predictors for early-achieved LLDAS when developing personalized management strategies for SLE, as well as in assisting physicians in managing SLE patients undergoing treatment with BAFF/APRIL inhibitors.

To the best of our knowledge, this study represents the first attempt to concurrently describe the cumulative probability of early-achieved LLDAS in SLE patients treated with BAFF/APRIL inhibitors telitacicept and belimumab. Consistent with real-life observational studies conducted in other populations (16, 17), our findings further validate the effectiveness of BAFF/APRIL inhibitors in facilitating the attainment of therapeutic targets. Specifically, we report an overall cumulative probability of 44.43% for early-achieved LLDAS, surpassing rates reported among SLE patients receiving standard treatment (6, 7). Among the patients who initially achieved LLDAS within 24 weeks of treatment, a total of 16.28% (7/43) subsequently lost LLDAS during the follow-up period. Despite demonstrating the dynamic nature of this condition, the overall rate of achieving and maintaining LLDAS appears to remain stable. The data suggests that the achievement of minimal disease activity, as per the LLDAS criteria, would be an appropriate goal in implementing a treat-to-target strategy, particularly during the early stages of the disease. In the univariable and multivariable analyses, active SLE patients have been found to be more likely to achieve an early-achieved LLDAS response with telitacicept treatment compared with belimumab, on the premise that there were few differences in baseline clinical characteristics between the two groups. The positive effect remained consistent across all subgroup analyses, and this conclusion was drawn based on the minimal differences in baseline clinical characteristics observed between the two groups. In this study, the frequency of early-achieved LLDAS attainment over time was 64.29% for those receiving telitacicept therapy and 35.56% for those receiving belimumab therapy. The observed frequency of LLDAS with belimumab aligns with the findings from a real-world study conducted in China, which reported a 35.71% achievement rate at 6 months (13). Additionally, previous randomized clinical trials evaluating belimumab have reported cumulative frequencies of LLDAS ranging from 12.5% to 14.4% over a period of 52 weeks (12). The primary reason for the observed discrepancy, as we hypothesized, can be attributed to the selection bias of SLE patients in the BLISS-52 (18) and BLISS-76 (19) trials, from which LLDAS was derived based on a relatively elevated baseline disease activity. In addition, the efficacy of telitacicept has been investigated in lupus patients, following its approval as a BAFF/APRIL inhibitor for the treatment of this patient population in China in March 2021. The superiority of telitacicept over belimumab has been documented in terms of SIR-4 response; however, it should be noted that the efficacy analyses were limited to this single outcome (SRI-4) (9, 11, 20–22). The available real-world data supporting the use of telitacicept in patients with SLE are still limited, and there remains a research gap regarding the frequency of achieving LLDAS in SLE patients (4). Besides, the disparity in LLDAS achievement between these two agents in actual clinical practice remains unclarified. Consistent with previously published data, the disease activity decreased in SLE patients following the addition of telitacicept therapy, indicating the significant efficacy of telitacicept in clinical practice. In a study conducted by H Jin (23), it was observed that patients receiving belimumab exhibited a significantly lower reduction in SLEDAI-2K and PGA at 24 weeks compared to those receiving telitacicept (P<0.05). And there were comparable elevations in C_3_ and C_4_ levels at 24 weeks between the two groups. Notably, our study did not observe any significant differences between telitacicept and belimumab regarding the decline in disease activity (SLEDAI-2K and PGA) or improvement in serologic markers. However, patients treated with telitacicept exhibited a significantly higher rate of response to LLDAS compared to those with belimumab.

An analysis of the failure to achieve LLDAS after 24 weeks of belimumab therapy initiation revealed that the most common reasons were meeting criterion 1 (SLEDAI-2K ≤4, with no evidence of activity in any major organ) and criterion 4 (current daily prednisone dosage ≤ 7.5mg). Consistent with previous studies, inadequate reduction in prednisolone dosage was identified as an unmet criterion for patients who did not achieve LLDAS (7). Although daily prednisolone dosage >7.5mg as a controversial cutoff to define low disease activity, it is important to consider that patients on high dosages of prednisolone may face increased risks of osteoporosis, infections, metabolic disorders, and cardiovascular disorders (24, 25). Therefore, it is acceptable to establish a lower cutoff during initial treatment for patients with SLE. We similarly found that the difference in LLDAS attainment between belimumab and telitacicept was greater in criterion 1 and criterion 4. The results suggested that telitacicept may take less time than belimumab for corticosteroid dose reduction and disease activity control in SLE treatment. Further analysis was conducted to examine the disparities in baseline characteristics between the LLDAS and N-LLDAS groups of telitacicept and belimumab, aiming to elucidate potential factors contributing to the patients’ failure to meet various criteria. The results of our study demonstrate that patients receiving belimumab treatment who did not achieve LLDAS exhibited significantly elevated disease activity (SLEDAI-2K, Clinical SLEDAI-2K), higher rates of renal and hematological involvement, as well as a greater proportion of hypocomplementemia at baseline. Conversely, these associations were not observed in patients receiving telitacicept treatment. Moreover, the individual analysis of 17 patients who did not meet both criterion 1 and criterion 4 for LLDAS on belimumab treatment similarly demonstrated prominent clinical characteristics, including elevated disease activity, reduced levels of serum albumin and complement, as well as increased renal and hematological involvement. It seems that the decline in LLDAS achievement over time of belimumab when compared to telitacicept, appears to be attributed to the inferior control of belimumab in SLE patients with high disease activity, renal and hematologic involvement, and hypocomplementemia. Telitacicept distinguishes itself from belimumab by inhibiting the binding of BAFF and APRIL to B cell receptors, particularly targeting the high affinity bond between APRIL and BCMA. The inhibition of the interaction between APRIL and BCMA has demonstrated efficacy in suppressing plasmablasts and long-lived plasma cells survival, as well as autoantibody secretion (26, 27). The disparity in initial disease amelioration between telitacicept and belimumab in treatment of SLE may be elucidated by this observation. No study has reported a superior benefit of telitacicept over belimumab in terms of response to LLDAS. Our data supports the significant effectiveness of BAFF/APRIL inhibitors in clinical practice, with lupus patients appearing to derive greater benefits from telitacicept compared to belimumab in early-achieved LLDAS. Collectly, our data supports the significant effectiveness of BAFF/APRIL inhibitors in clinical practice, with lupus patients appearing to derive greater benefits from telitacicept compared to belimumab in early-achieved LLDAS.

Among the predictors identified in our cohort, independent risk factors for failure to attain early-achieved LLDAS included low lymphocyte counts, decline in serum albumin levels, and hematological involvement. The SLICC cohort study revealed that lower levels of SLEDAI and prednisone were significantly associated with achieving LLDAS at 12 months (28). In our study, univariate analysis results indicated a significant association between lower SLEDAI-2K levels and early attainment of LLDAS; however, this significance disappeared in the multivariate analysis. Considering that disease activity influences treatment decisions, the use of immunosuppressant drugs was found to be a predictor of failure to achieve LLDAS (4). Therefore, it would be reasonable to emphasize treatment as a predictor after adjusting for SLEDAI-2K. According to previous studies (6, 7, 29), there was a negative association between renal involvement and hematological involvement with the achievement of LLDAS. In our study, significant differences were observed in baseline characteristics between patients who achieved LLDAS and those who did not, particularly in terms of baseline albumin levels. Cox regression analyses revealed that albumin level, as a specific indicator of renal involvement, emerged as a significant marker for subsequent attainment of LLDAS. Renal involvement is one of the most prevalent major organ involvements among Chinese SLE patients, contributing to increased clinical complexity and higher disease activity scores (2, 30). Our data further underscores the crucial role played by renal and hematological involvement in influencing the early achievement of LLDAS.

The decreased lymphocyte counts were found to be predictive of failure in achieving LLDAS in this population, which highlights their significance as a prognostic indicator. The absolute counts of lymphocytes reflect their proliferation ability, and in recent years, researchers have increasingly utilized the absolute counts of lymphocyte subsets as an important immune indicator for assessing the prognosis and efficacy of tumor patients (31). In SLE patients, lymphopenia is recognized as the most common white blood cell abnormality, with most leucopenia episodes being driven by lymphopenia (32). Previous studies have demonstrated a close association between the absolute counts of lymphocytes and SLE disease activity; frequent decreases in peripheral lymphocytes occur among critical and active SLE patients (33). Our study has yielded the following findings: Firstly, a significant association was observed between lower lymphocyte counts and an increased risk of failing to achieve early-achieved LLDAS. This suggests that patients with reduced lymphocyte counts may not experience rapid improvement through BAFF/APRIL intervention. Interestingly, our results contradict the findings of Ramsköld et al (34), who suggested that low baseline lymphocyte counts predict response to belimumab treatment. Similarly, their results also indicated that low B-cell counts have predictive value. It is widely recognized that adaptive immunity plays a pivotal role in the pathogenesis of SLE, wherein an increased population of autoreactive mature naive B cells can subsequently differentiate into plasma cells producing autoantibodies (35, 36). Therefore, it is reasonable to consider assessing B-cell counts as a valuable parameter at the initiation of belimumab treatment. In contrast, the study by Francesca et al. (37) demonstrated that the baseline percentage of CD8+ effector memory T cells was associated with SLEDAI-2K and its subsequent improvement after 12 months of belimumab therapy. Lymphopenia, mainly characterized by low absolute counts of B and T cells, presents complex pathophysiological mechanisms and an unclear etiopathogenesis (38, 39). Current B-cell therapies demonstrate efficacy in SLE; however, they may prove inadequate for patients with refractory SLE characterized by potent non-BAFF/APRIL-dependent pathogenic pathways (40–42). Consequently, we considered whether certain mechanisms of lymphocytopenia could be associated with suboptimal disease control in SLE patients on BAFF/APRIL inhibitors treatment. This discovery highlights the necessity and significance of monitoring lymphocyte counts in SLE patients undergoing treatment with BAFF/APRIL inhibitors, facilitating comprehension of immunologic damage in lupus patients, analysis of clinical conditions, and prediction of therapeutic outcomes. However, further investigations are warranted to assess the potential mechanisms underlying the association between lymphocyte subsets and response to this therapeutic approach: among other plausible explanations, exploration of BAFF/APRIL-independent pathways governing B-cell survival, examination of endogenous BAFF-neutralizing autoantibodies, or investigation into the emergence of anti-drug antibodies would be intriguing avenues to explore.

Since each patient’s negative predictors of LLDAS are probably unique and complex, it impractical to predict LLDAS solely based on a single variable. Establishing prognostic stratification using multiple risk factors would be a more viable and reliable approach. The risk stratification in our study was based on the cumulative number of risk factors, and the findings demonstrate a significant inverse correlation between the frequency of early-achieved LLDAS and the number of risk factors in patients with SLE. This emphasizes the importance of employing diverse risk indicators to accurately predict a patient’s likelihood for early achievement of LLDAS.

The overall tolerability of Telitacicept and belimumab in Chinese lupus patients was satisfactory. A total of 17 (18.39%) patients experienced 19 AEs during the study period. The majority of these AEs were mild and well-tolerated. In terms of specific AEs, viral infections and urinary tract infections were the most commonly reported infection-related events in our study cohort, which aligns with findings from other real-world studies conducted in China (13). However, it is important to exercise caution when interpreting these results due to potential limitations associated with our observational study design.

Our study has several limitations that need to be acknowledged. Firstly, our analyses were limited to a single outcome (LLDAS) within 24 weeks. While early achievement of LLDAS is a pivotal measure for disease modification in SLE, considering multiple outcomes would offer a more comprehensive perspective on efficacy and provide a nuanced understanding of telitacicept’s potential in attaining LLDAS compared to belimumab. Conducting additional analyses at different timepoints would be valuable for gaining insights into the rapidity of treatment effectiveness and the durability of efficacy. Secondly, the retrospective study design posed challenges in dealing with missing data within the real-life cohort, thereby impacting our analysis by excluding potential factors that were infrequently considered. Thirdly, the observational design of our study made it difficult to accurately assess the influence of treatment in an unselected heterogeneous cohort. The therapy decisions were based on individual disease severity and specific damage, further complicating the exclusion of treatment effects. Additionally, due to a limited number of patients enrolled in our study, we faced hindrances in identifying additional risk factors and developing a more comprehensive predictive model. While our study makes valuable contributions to the field, it is important to consider its limitations. Future research should focus on utilizing prospective study designs, increasing sample sizes, and using qualified data from other centers for external validation to improve the accuracy and applicability of our findings in clinical practice.

In conclusion, our study has provided valuable data on the impact of biologics in achieving LLDAS and compared the differential effects between telitacicept and belimumab. The consistent positive effect of telitacicept administration on early attainment of LLDAS was observed. Furthermore, we successfully identified hematological involvement, baseline lymphocyte counts and serum albumin levels as significant predictors for early achievement of LLDAS.

## Data availability statement

The raw data supporting the conclusions of this article will be made available by the authors, without undue reservation.

## Ethics statement

The studies involving humans were approved by the Central Ethics Committee of Nanfang Hospital, Southern Medical University. The studies were conducted in accordance with the local legislation and institutional requirements. The participants provided their written informed consent to participate in this study. Written informed consent was obtained from the individual(s), and minor(s)’ legal guardian/next of kin, for the publication of any potentially identifiable images or data included in this article.

## Author contributions

CF: Conceptualization, Writing – review & editing, Data curation, Formal analysis, Methodology, Project administration, Software, Visualization, Writing – original draft. TY: Writing – original draft, Data curation. SZ: Writing – review & editing, Writing – original draft, Conceptualization, Formal analysis. XL: Writing – original draft, Writing – review & editing. RX: Data curation, Writing – original draft. SC: Supervision, Writing – review & editing, Writing – original draft. JL: Conceptualization, Supervision, Writing – review & editing.
