# Supplementary material for: Frequency and predictors for early-achieved lupus low disease activity state in systemic lupus erythematosus patients treated with telitacicept or belimumab: A real-life, single-center observational study
Source: Front Immunol. 2024 Jun 14;15:1423035. doi: 10.3389/fimmu.2024.1423035 (PMC11211586; doi:10.3389/fimmu.2024.1423035)
Supplement: Supplementary file 1 [file DataSheet_1.docx]

***Supplementary Material***

**Supplementary Table S1.** Variable coefficients obtain according to the selected best penalty parameter(λ)

| Variable | Coefficients |
| --- | --- |
| SLEDAI-2K^1^ | 0 |
| Clinical SLEDAI-2K^2^ | 0 |
| Hematological |  |
| No | 0.657713109 |
| Yes | -0.003622365 |
| Renal |  |
| No | 0 |
| Yes | 0 |
| Hypocomplementemia |  |
| No | 0 |
| Yes | 0 |
| Anti-SSB |  |
| No | -0.730570788 |
| Yes | 0 |
| Anti-Ro52 |  |
| No | -0.903737052 |
| Yes | 2.68779E-15 |
| WBC^3^ | 0 |
| C_3_^4^ | 0 |
| Lymphocyte counts | 1.231035089 |
| Serum albumin | 0.062404449 |
| BAFF/APRIL inhibitors |  |
| belimumab | -1.515797847 |
| telitacicept | 6.29438E-15 |

^1^SLEDAI-2K=Systemic Lupus Erythematosus Disease Activity Index 2000

^2^Clinical SLEDAI-2K=SLEDAI-2K excluding Anti-dsDNA and complement scores

^3^WBC=white blood cell

^4^C_3_=complement C_3_

**Supplementary Table S2.** Comparison of baseline clinical and serological characteristics in patients treated with belimumab or telitacicept.

| Variable | Belimumab1 | Telitacicept1 | P value |
| --- | --- | --- | --- |
|  | N=45 | N=42 |  |
| Gender(female) | 39 (86.67) | 37 (88.10) | 0.841 |
| Age(years) | 30.00 (23.00-36.00) | 28.50 (20.50-35.00) | 0.434 |
| Age at diagnosis(years) | 22.00 (17.00-29.00) | 21.50 (15.00-28.00) | 0.372 |
| Disease duration(years) | 48.00 (12.00-133.00) | 43.50 (7.25-123.25) | 0.69 |
| Disease activity |  |  |  |
| SLEDAI-2K | 10.00 (6.00-14.00) | 8.00 (5.00-12.75) | 0.571 |
| Clinical SLEDAI-2K | 6.00 (4.00-10.00) | 6.00 (3.00-9.75) | 0.483 |
| PGA | 1.20 (0.60-1.60) | 0.80 (0.50-1.60) | 0.548 |
| Organ involvement |  |  |  |
| Gastrointestinal | 0 (0.00) | 4 (9.52) | 0.05 |
| Neurological | 2 (4.44) | 5 (11.90) | 0.255 |
| Cardiovascular/respiratory | 7 (15.56) | 3 (7.14) | 0.219 |
| APS | 6 (13.33) | 6 (14.29) | 0.898 |
| Musculoskeletal | 20 (44.44) | 12 (28.57) | 0.125 |
| Mucocutaneous | 29 (64.44) | 26 (61.90) | 0.806 |
| Hematological | 21 (46.67) | 21 (50.00) | 0.756 |
| Renal | 21 (46.67) | 15 (35.71) | 0.3 |
| Constitutional | 12 (26.67) | 11 (26.19) | 0.96 |
| Serosal | 1 (2.22) | 8 (19.05) | **0.013** |
| Serologic |  |  |  |
| Hypocomplementemia | 23 (51.11) | 23 (54.76) | 0.733 |
| C3（g /L） | 0.72 (0.57-0.94) | 0.70 (0.53-0.96) | 0.875 |
| C4（g /L） | 0.15 (0.08-0.19) | 0.14 (0.08-0.22) | 0.983 |
| Anti-dsDNA | 30 (66.67) | 27 (64.29) | 0.815 |
| Anti-dsDNA（μ /mL） | 127.55 (26.18-333.58) | 179.45 (19.21-482.36) | 0.552 |
| Medications |  |  |  |
| Untreated | 14 (31.11) | 12 (28.57) | 0.796 |
| Daily prednisone (mg/d) | 20.00 (10.00-45.00) | 27.50 (7.50-43.75) | 0.911 |
| Mycophenolate | 26 (57.78) | 23 (54.76) | 0.777 |
| Azathioprine | 4 (8.89) | 2 (4.76) | 0.677 |
| Methotrexate | 1 (2.22) | 2 (4.76) | 0.608 |
| Leflunomide | 4 (8.89) | 0 (0.00) | 0.117 |
| Tacrolimus | 2 (4.44) | 3 (7.14) | 0.669 |
| Ciclosporin | 9 (20.00) | 7 (16.67) | 0.688 |
| HCQ | 43 (95.56) | 40 (95.24) | 1 |
| ANA | 44 (97.78) | 42 (100.00) | 1 |
| ANA（μ /mL） | 333.76 (105.11-427.10) | 222.89 (53.72-356.13) | 0.067 |
| Anti-SSA | 23 (51.11) | 23 (54.76) | 0.733 |
| Anti-SSB | 7 (15.56) | 6 (14.29) | 0.868 |
| Anti-Sm | 15 (33.33) | 16 (38.10) | 0.643 |
| Anti-U1RNP | 28 (62.22) | 27 (64.29) | 0.842 |
| ARPA/Rib-P | 17 (37.78) | 13 (30.95) | 0.503 |
| AnuA | 22 (48.89) | 20 (47.62) | 0.906 |
| AHA | 21 (46.67) | 20 (47.62) | 0.929 |
| AMAM2 | 4 (8.89) | 8 (19.05) | 0.17 |
| Anti-Ro52 | 23 (51.11) | 18 (42.86) | 0.441 |
| Anti-β2GP1 | 7 (15.56) | 12 (28.57) | 0.142 |
| ACA | 2 (4.44) | 8 (19.05) | **0.033** |
| Proteinuria | 23 (51.11) | 16 (38.10) | 0.223 |
| WBC（× 109 /L） | 4.51 (3.63-7.51) | 5.42 (4.38-6.73) | 0.53 |
| Lymphocyte counts（× 109 /L） | 1.21 (0.84-2.07) | 1.38 (1.04-1.82) | 0.986 |
| Neutrophils counts（× 109 /L） | 3.20 (2.19-4.64) | 3.38 (2.53-4.16) | 0.64 |
| Monocyte counts（× 109 /L） | 0.42 (0.27-0.58) | 0.43 (0.29-0.56) | 0.753 |
| HGB（g /L） | 120.00 (100.00-132.00) | 114.00 (97.25-131.00) | 0.724 |
| PLT（× 109 /L） | 223.00 (178.00-277.00) | 226.00 (165.75-264.00) | 0.546 |
| IgA（g /L） | 2.56 (1.97-3.58) | 2.41 (1.63-3.57) | 0.519 |
| IgM（g /L） | 0.91 (0.67-1.26) | 0.80 (0.55-0.95) | 0.056 |
| IgG（g /L） | 13.44 (10.12-17.75) | 13.08 (10.05-15.53) | 0.728 |
| CH50（g /L） | 35.00 (21.00-43.00) | 33.25 (20.25-44.00) | 0.715 |
| ESR（mm /H） | 20.00 (12.00-40.00) | 31.00 (14.00-51.50) | 0.439 |
| CRP（mg /L） | 1.08 (0.48-2.72) | 2.64 (1.00-5.13) | **0.016** |
| Serum albumin（g /L） | 34.40 (32.30-39.60) | 37.50 (31.32-39.93) | 0.99 |
| Globulin（g /L） | 28.90 (24.30-34.60) | 27.35 (24.32-31.80) | 0.825 |

Clinical SLEDAI-2K=SLEDAI-2K excluding Anti-dsDNA and complement scores; PGA= Physician’s Global Assessment; APS=anticardiolipin syndrome; C_3_=complement C_3_; C_4_=complement C_4_; HCQ= hydroxychloroquine; WBC=white blood cell; HGB=hemoglobin; PLT= platelet; Ig=immunoglobulin; CH_50_=50% hemolytic unit of complement; CRP=C-reactive protein; ESR=erythrocyte sedimentation rat; ^1^N (%) or median (IQR); Values in bold indicate that P values < 0.05.

**Supplementary Table S3.** Baseline demographic, clinical and serological characteristics in patients treated with telitacicept.

| Variable | N-LLDAS^1^ | LLDAS^1^ | P value |
| --- | --- | --- | --- |
|  | N=15 | N=27 |  |
| Gender(female) | 27 (93.10) | 12 (75.00) | 0.166 |
| Age(years) | 25.00 (20.50-32.50) | 32.00 (21.00-36.00) | 0.415 |
| Age at diagnosis(years) | 22.00 (15.00-27.00) | 21.00 (15.00-28.00) | 0.742 |
| Disease duration(years) | 65.00 (10.50-98.00) | 30.00 (6.50-155.50) | 0.864 |
| Disease activity |  |  |  |
| SLEDAI-2K | 10.00 (7.50-17.00) | 6.00 (4.00-12.00) | 0.206 |
| Clinical SLEDAI-2K | 6.00 (4.00-14.00) | 6.00 (2.50-8.50) | 0.335 |
| PGA | 1.20 (0.75-2.25) | 0.60 (0.45-1.50) | 0.201 |
| Organ involvement |  |  |  |
| Gastrointestinal | 2 (13.33) | 2 (7.41) | 1 |
| Neurological | 1 (6.67) | 4 (14.81) | 0.639 |
| Cardiovascular/respiratory | 3 (20.00) | 0 (0.00) | **0.040** |
| APS | 3 (20.00) | 3 (11.11) | 0.649 |
| Musculoskeletal | 2 (13.33) | 10 (37.04) | 0.103 |
| Mucocutaneous | 10 (66.67) | 16 (59.26) | 0.636 |
| Hematological | 10 (66.67) | 11 (40.74) | 0.107 |
| Renal | 7 (46.67) | 8 (29.63) | 0.27 |
| Constitutional | 4 (26.67) | 7 (25.93) | 0.958 |
| Serosal | 4 (26.67) | 4 (14.81) | 0.425 |
| Serologic |  |  |  |
| Hypocomplementemia | 10 (66.67) | 13 (48.15) | 0.248 |
| C_3_（g /L） | 0.64 (0.52-0.90) | 0.74 (0.54-1.06) | 0.358 |
| C_4_（g /L） | 0.10 (0.08-0.21) | 0.14 (0.08-0.22) | 0.581 |
| Anti-dsDNA | 11 (73.33) | 16 (59.26) | 0.362 |
| Anti-dsDNA（μ /mL） | 492.30 (53.19-591.62) | 90.94 (14.39-420.11) | 0.168 |
| Medications |  |  |  |
| Untreated | 5 (33.33) | 7 (25.93) | 0.611 |
| Daily prednisone (mg/d) | 30.00 (10.00-45.00) | 15.00 (5.00-42.50) | 0.307 |
| Mycophenolate | 10 (66.67) | 13 (48.15) | 0.248 |
| Azathioprine | 0 (0.00) | 2 (7.41) | 0.53 |
| Methotrexate | 1 (6.67) | 1 (3.70) | 1 |
| Leflunomide | 0 (0.00) | 0 (0.00) | 1 |
| Tacrolimus | 2 (13.33) | 1 (3.70) | 0.287 |
| Ciclosporin | 2 (13.33) | 5 (18.52) | 1 |
| HCQ | 15 (100.00) | 25 (92.59) | 0.53 |
| ANA | 15 (100.00) | 27 (100) | 1 |
| ANA（μ /mL） | 63.16 (25.81-227.55) | 265.99 (134.84-407.23) | **0.035** |
| Anti-SSA | 6 (40.00) | 17 (62.96) | 0.152 |
| Anti-SSB | 2 (13.33) | 4 (14.81) | 1 |
| Anti-Sm | 6 (40.00) | 10 (37.04) | 0.85 |
| Anti-U1RNP | 9 (60.00) | 18 (66.67) | 0.666 |
| ARPA/Rib-P | 5 (33.33) | 8 (29.63) | 0.804 |
| AnuA | 7 (46.67) | 13 (48.15) | 0.927 |
| AHA | 7 (46.67) | 13 (48.15) | 0.927 |
| AMAM2 | 1 (6.67) | 7 (25.93) | 0.222 |
| Anti-Ro52 | 4 (26.67) | 14 (51.85) | 0.114 |
| Anti-β2GP1 | 5 (33.33) | 7 (25.93) | 0.611 |
| ACA | 4 (26.67) | 4 (14.81) | 0.425 |
| Proteinuria | 7 (46.67) | 9 (33.33) | 0.394 |
| WBC（× 10^9^ /L） | 4.76 (3.67-5.90) | 5.49 (5.08-7.25) | **0.042** |
| Lymphocyte counts（× 10^9^ /L） | 1.03 (0.63-1.35) | 1.52 (1.27-1.94) | **0.002** |
| Neutrophils counts（× 10^9^ /L） | 3.14 (2.21-3.87) | 3.58 (2.88-4.71) | 0.222 |
| Monocyte counts（× 10^9^ /L） | 0.42 (0.22-0.50) | 0.44 (0.32-0.60) | 0.408 |
| HGB（g /L） | 106.00 (96.00-120.00) | 120.00 (102.00-134.50) | 0.115 |
| PLT（× 10^9^ /L） | 205.00 (183.00-257.50) | 243.00 (157.00-267.50) | 0.646 |
| IgA（g /L） | 2.32 (1.64-3.21) | 2.59 (1.66-3.64) | 0.753 |
| IgM（g /L） | 0.70 (0.54-0.86) | 0.81 (0.58-0.99) | 0.282 |
| IgG（g /L） | 9.94 (8.99-13.31) | 13.61 (11.71-15.72) | **0.021** |
| CH_50_（g /L） | 29.00 (19.00-34.90) | 36.00 (20.50-48.95) | 0.202 |
| ESR（mm /H） | 33.90 (22.50-67.00) | 24.00 (9.50-40.50) | 0.101 |
| CRP（mg /L） | 2.33 (1.06-6.90) | 2.95 (0.94-5.13) | 0.599 |
| Serum albumin（g /L） | 33.10 (28.45-38.60) | 38.10 (32.15-40.40) | 0.098 |
| Globulin（g /L） | 25.60 (23.10-32.50) | 28.50 (26.00-31.50) | 0.416 |

LLDAS=lupus low disease activity state; N-LLDAS=non-LLDAS; Clinical SLEDAI-2K=SLEDAI-2K excluding Anti-dsDNA and complement scores; PGA= Physician’s Global Assessment; APS=anticardiolipin syndrome; C_3_=complement C_3_; C_4_=complement C_4_; HCQ= hydroxychloroquine; WBC=white blood cell; HGB=hemoglobin; PLT= platelet; Ig=immunoglobulin; CH50=50% hemolytic unit of complement; CRP=C-reactive protein; ESR=erythrocyte sedimentation rat; ^1^N (%) or median (IQR); Values in bold indicate that P values < 0.05.

**Supplementary Table S4.** Baseline demographic, clinical and serological characteristics in patients treated with belimumab.

| Variable | N-LLDAS^1^ | LLDAS^1^ | P value |
| --- | --- | --- | --- |
|  | N=29 | N=16 |  |
| Gender(female) | 27 (93.10) | 12 (75.00) | 0.166 |
| Age(years) | 30.00 (24.00-36.00) | 30.00 (22.75-36.00) | 0.678 |
| Age at diagnosis(years) | 23.00 (16.00-28.00) | 21.00 (17.00-34.00) | 0.669 |
| Disease duration(years) | 78.00 (22.00-137.00) | 32.00 (7.25-89.75) | 0.162 |
| Disease activity |  |  |  |
| SLEDAI-2K | 12.00 (6.00-14.00) | 7.00 (4.00-11.50) | **0.046** |
| Clinical SLEDAI-2K | 8.00 (5.00-11.00) | 5.00 (4.00-9.00) | **0.046** |
| PGA | 1.40 (0.60-2.00) | 0.80 (0.40-1.30) | 0.13 |
| Organ involvement |  |  |  |
| Gastrointestinal | 0 (0.00) | 0 (0.00) | 1 |
| Neurological | 1 (3.45) | 1 (6.25) | 1 |
| Cardiovascular/respiratory | 5 (17.24) | 2 (12.50) | 1 |
| APS | 5 (17.24) | 1 (6.25) | 0.399 |
| Musculoskeletal | 13 (44.83) | 7 (43.75) | 0.944 |
| Mucocutaneous | 17 (58.62) | 12 (75.00) | 0.272 |
| Hematological | 17 (58.62) | 4 (25.00) | **0.030** |
| Renal | 17 (58.62) | 4 (25.00) | **0.030** |
| Constitutional | 7 (24.14) | 5 (31.25) | 0.606 |
| Serosal | 1 (3.45) | 0 (0.00) | 1 |
| Serologic |  |  |  |
| Hypocomplementemia | 18 (62.07) | 5 (31.25) | **0.048** |
| C_3_（g /L） | 0.68 (0.52-0.84) | 0.83 (0.69-1.06) | 0.05 |
| C_4_（g /L） | 0.11 (0.06-0.17) | 0.16 (0.13-0.23) | 0.117 |
| Anti-dsDNA | 21 (72.41) | 9 (56.25) | 0.271 |
| Anti-dsDNA（μ /mL） | 137.85 (48.22-367.10) | 99.31 (22.92-225.10) | 0.162 |
| Medications |  |  |  |
| Untreated | 9 (31.03) | 5 (31.25) | 0.988 |
| Daily prednisone (mg/d) | 20.00 (10.00-45.00) | 17.50 (6.88-45.00) | 0.503 |
| Mycophenolate | 15 (51.72) | 11 (68.75) | 0.268 |
| Azathioprine | 3 (10.34) | 1 (6.25) | 1 |
| Methotrexate | 0 (0.00) | 1 (6.25) | 0.356 |
| Leflunomide | 2 (6.90) | 2 (12.50) | 0.608 |
| Tacrolimus | 2 (6.90) | 0 (0.00) | 0.531 |
| Ciclosporin | 7 (24.14) | 2 (12.50) | 0.456 |
| HCQ | 29 (100.00) | 14 (87.50) | 0.121 |
| ANA | 29 (100.00) | 15 (93.8) | 0.356 |
| ANA（μ /mL） | 332.78 (135.73-499.48) | 364.87 (82.60-407.70) | 0.602 |
| Anti-SSA | 15 (51.72) | 8 (50.00) | 0.912 |
| Anti-SSB | 1 (3.45) | 6 (37.50) | **0.005** |
| Anti-Sm | 10 (34.48) | 5 (31.25) | 0.826 |
| Anti-U1RNP | 18 (62.07) | 10 (62.50) | 0.977 |
| ARPA/Rib-P | 12 (41.38) | 5 (31.25) | 0.502 |
| AnuA | 18 (62.07) | 4 (25.00) | **0.017** |
| AHA | 15 (51.72) | 6 (37.50) | 0.36 |
| AMAM2 | 4 (13.79) | 0 (0.00) | 0.281 |
| Anti-Ro52 | 12 (41.38) | 11 (68.75) | 0.079 |
| Anti-β2GP1 | 6 (20.69) | 1 (6.25) | 0.393 |
| ACA | 2 (6.90) | 0 (0.00) | 0.531 |
| Proteinuria | 17 (58.62) | 6 (37.50) | 0.175 |
| WBC（× 10^9^ /L） | 4.47 (3.62-6.83) | 6.27 (4.02-8.04) | 0.151 |
| Lymphocyte counts（× 10^9^ /L） | 1.12 (0.72-1.58) | 1.97 (1.35-2.70) | **0.002** |
| Neutrophils counts（× 10^9^ /L） | 3.09 (2.19-4.18) | 4.17 (2.17-4.92) | 0.407 |
| Monocyte counts（× 10^9^ /L） | 0.41 (0.26-0.54) | 0.47 (0.28-0.63) | 0.25 |
| HGB（g /L） | 113.00 (98.00-132.00) | 120.50 (110.50-130.00) | 0.514 |
| PLT（× 10^9^ /L） | 223.00 (187.00-251.00) | 221.00 (176.00-298.00) | 0.661 |
| IgA（g /L） | 2.62 (2.13-3.58) | 2.35 (1.93-3.59) | 0.722 |
| IgM（g /L） | 0.87 (0.67-1.16) | 0.91 (0.74-1.36) | 0.635 |
| IgG（g /L） | 13.44 (10.03-16.40) | 13.36 (10.34-18.72) | 0.831 |
| CH_50_（g /L） | 32.20 (18.00-39.00) | 38.50 (24.75-46.75) | 0.145 |
| ESR（mm /H） | 31.00 (14.00-50.00) | 17.50 (8.00-36.00) | 0.12 |
| CRP（mg /L） | 1.74 (0.48-3.24) | 0.68 (0.47-1.03) | 0.123 |
| Serum albumin（g /L） | 34.30 (31.10-38.90) | 36.95 (33.72-41.40) | 0.104 |
| Globulin（g /L） | 28.90 (23.80-33.10) | 28.85 (24.30-36.32) | 0.731 |

LLDAS=lupus low disease activity state; N-LLDAS=non-LLDAS; Clinical SLEDAI-2K=SLEDAI-2K excluding Anti-dsDNA and complement scores; PGA= Physician’s Global Assessment; APS=anticardiolipin syndrome; C_3_=complement C_3_; C_4_=complement C_4_; HCQ= hydroxychloroquine; WBC=white blood cell; HGB=hemoglobin; PLT= platelet; Ig=immunoglobulin; CH50=50% hemolytic unit of complement; CRP=C-reactive protein; ESR=erythrocyte sedimentation rat; ^1^N (%) or median (IQR); Values in bold indicate that P values < 0.05.

**Supplementary Table S5**. Adverse events.

| Adverse Events | N (%) |
| --- | --- |
| Upper respiratory infection | 3（3.4） |
| Herpes zoster infections | 6（5.7） |
| Urinary tract infection | 5（5.7） |
| HSV infections | 2（2.2） |
| Pneumonia * | 2（2.2） |
| cholecystitis | 1（1.1） |

* Pneumonia: all had bacterial pneumonia.


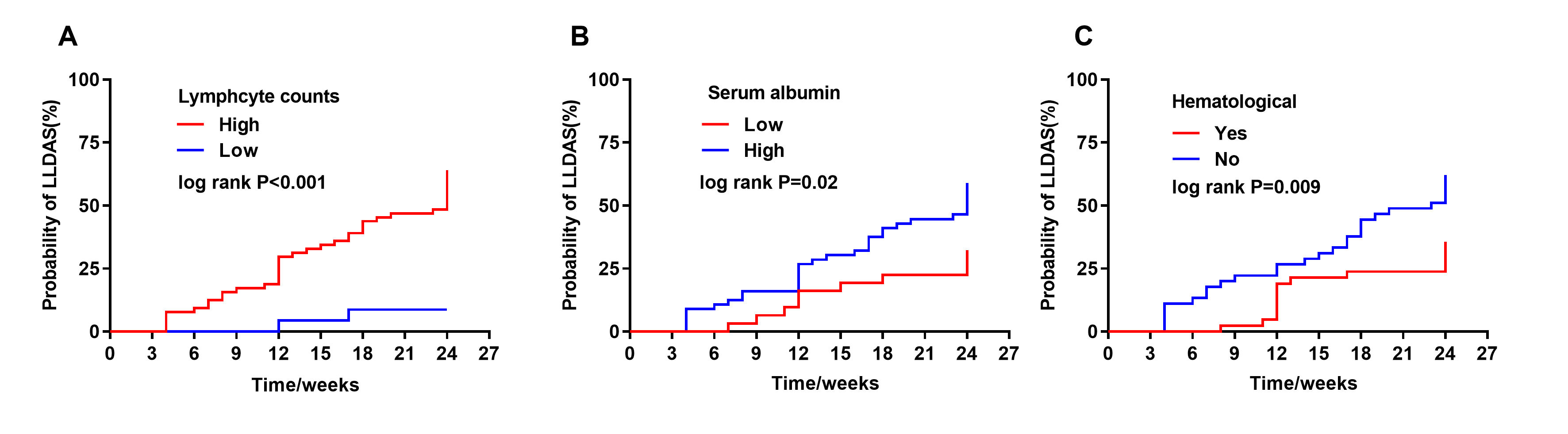


**Supplementary Figure S1 Follow-up information and survival analysis for LLDAS according to independent predictors in the cohort**. Kaplan-Meier curves illustrate the cumulative probability of LLDAS based on high/low lymphocyte counts (A), high/low serum albumin level (B), and presence or absence of hematological involvement (C).


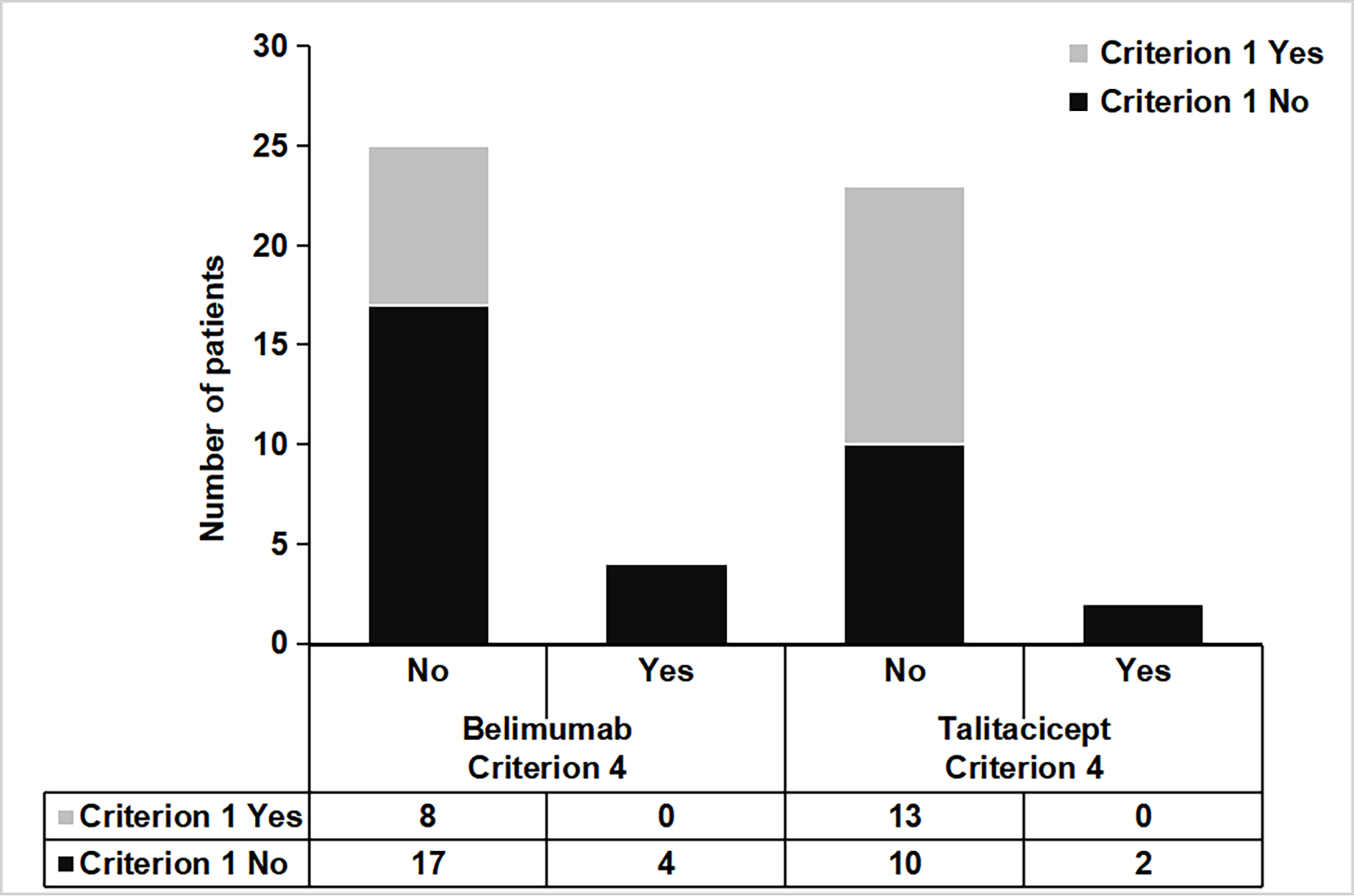


**Supplementary Figure S2 Analysis of criterion 1 and criterion 4 for LLDAS in patients treated with belimumab and telitacicept individually.**


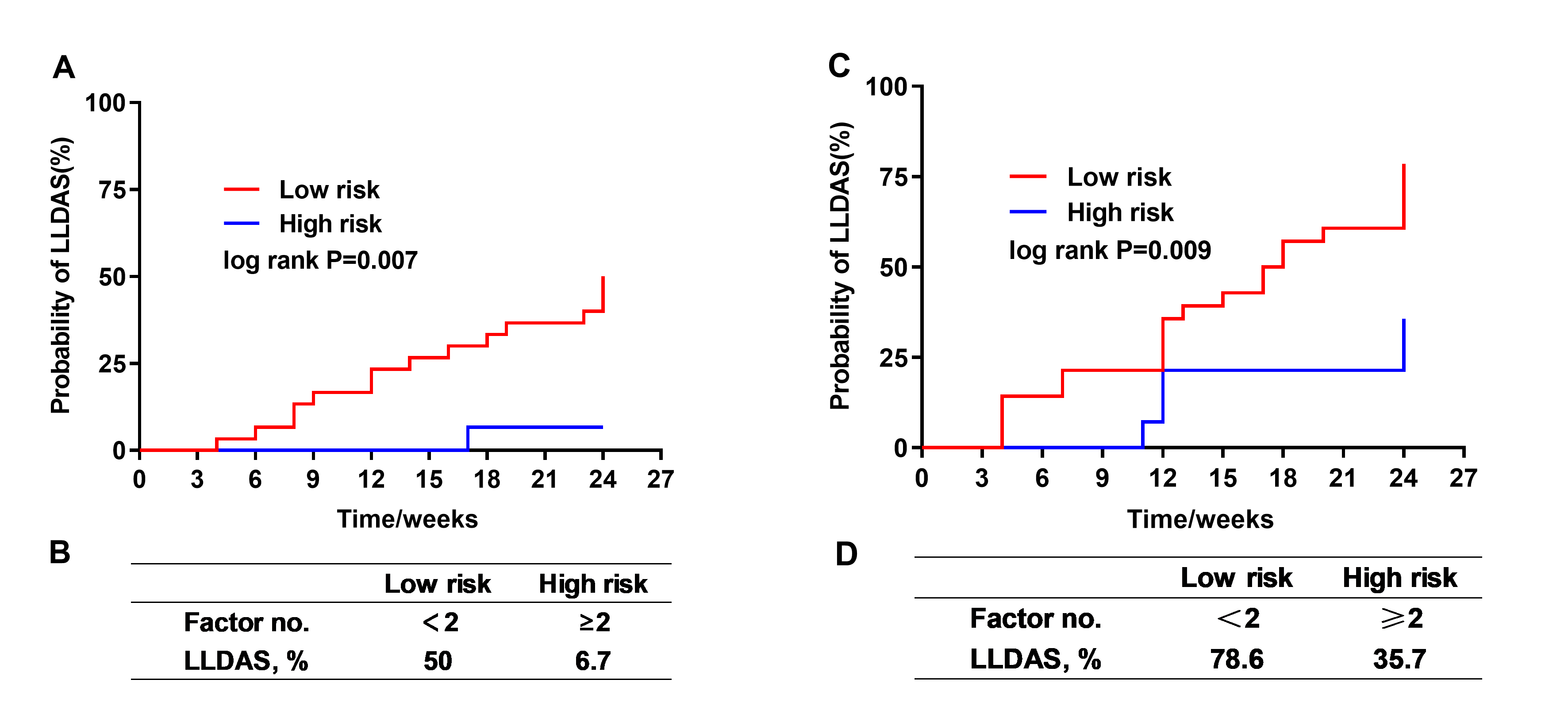


**Supplementary Figure S3 The prognostic stratification for LLDAS according to independent predictors after stratified analysis based on the use of BAFF/APRIL inhibitors.** The Kaplan-Meier estimations of LLDAS incidence were demonstrated among the two risk groups in patients treated with belimumab (A) or telitacicept (C). The probability of achieving LLDAS declined with an increasing number of risk factors in patients treated with belimumab (B) or telitacicept (D).

G
